# Serum influences the expression of *Pseudomonas aeruginosa* quorum-sensing genes and QS-controlled virulence genes during early and late stages of growth

**DOI:** 10.1002/mbo3.147

**Published:** 2013-12-23

**Authors:** Cassandra Kruczek, Uzma Qaisar, Jane A Colmer-Hamood, Abdul N Hamood

**Affiliations:** Department of Immunology and Molecular Microbiology, School of Medicine, Texas Tech University Health Sciences CenterLubbock, Texas, 79430

**Keywords:** *Pseudomonas aeruginosa*, quorum sensing, transcription regulation, virulence factors

## Abstract

In response to diverse environmental stimuli at different infection sites, *Pseudomonas aeruginosa*, a serious nosocomial pathogen, coordinates the production of different virulence factors through a complicated network of the hierarchical quorum-sensing (QS) systems including the *las*, *rhl*, and the 2-alkyl-4-quinolone-related QS systems. We recently showed that at early stages of growth serum alters the expression of numerous *P. aeruginosa* genes. In this study, we utilized transcriptional analysis and enzyme assays to examine the effect of serum on the QS and QS-controlled virulence factors during early and late phases of growth of the *P. aeruginosa* strain PAO1. At early phase, serum repressed the transcription of *lasI*, *rhlI*, and *pqsA* but not *lasR* or *rhlR*. However, at late phase, serum enhanced the expression of all QS genes. Serum produced a similar effect on the synthesis of the autoinducers 3OC_12_-HSL, C_4_-HSL, and HHQ/PQS. Additionally, serum repressed the expression of several QS-controlled genes in the early phase, but enhanced them in the late phase. Furthermore, serum influenced the expression of different QS-positive (*vqsR*, *gacA*, and *vfr*) as well as QS-negative (*rpoN*, *qscR*, *mvaT*, and *rsmA*) regulatory genes at either early or late phases of growth. However, with the exception of PAOΔ*vfr*, we detected comparable levels of *lasI*/*lasR* expression in PAO1 and PAO1 mutants defective in these regulatory genes. At late stationary phase, serum failed to enhance *lasI*/*lasR* expression in PAOΔ*vfr*. These results suggest that depending on the phase of growth, serum differentially influenced the expression of *P. aeruginosa* QS and QS-controlled virulence genes. In late phase, serum enhanced the expression of *las* genes through *vfr*.

## Introduction

*Pseudomonas aeruginosa* is a versatile Gram-negative opportunistic pathogen that survives under different environmental conditions (Lyczak et al. 2000). *Pseudomonas aeruginosa* causes a wide range of infections in immunocompromised individuals including human immunodeficiency virus (HIV)-infected patients and cancer patients undergoing chemotherapy (Driscoll et al. 2007; Pier and Ramphal 2010). *Pseudomonas aeruginosa* also causes serious infections in patients with acute or chronic wounds, individuals with cystic fibrosis (CF), and patients in intensive care units (Sadikot et al. 2005; Branski et al. 2009; Pier and Ramphal 2010). Damage produced by *P. aeruginosa* at different infection sites is due to the production of numerous cell-associated and extracellular virulence factors (van Delden 2004; Sadikot et al. 2005). Cell-associated virulence factors include pili, flagella, and lipopolysaccharide; extracellular virulence factors include exotoxin A, pyocyanin, proteases, and elastase (Lyczak et al. 2000; van Delden 2004; Sadikot et al. 2005; Pier and Ramphal 2010).

*Pseudomonas aeruginosa* coordinates the production of different virulence factors and biofilm formation through the cell density–dependent signaling system, quorum sensing (QS) (de Kievit and Iglewski 2000; Rumbaugh et al. 2000; Yoon et al. 2002; Hentzer et al. 2003; Smith and Iglewski 2003; Schuster and Greenberg 2006). The signaling is accomplished through diffusible *N*-acylhomoserine lactone (AHL) signal molecules termed autoinducers. *Pseudomonas aeruginosa* possesses two well-characterized QS systems, *las* and *rhl*, which are hierarchically arranged, with the entire QS cascade being controlled by the *las* system (Latifi et al. 1996; de Kievit and Iglewski 2000; Rumbaugh et al. 2000). Each system consists of a transcriptional activator (LasR, RhlR) and an autoinducer synthase (LasI, RhlI). LasI directs the synthesis of *N-*(3-oxododecanoyl)-l-homoserine lactone (3OC_12_-HSL), whereas RhlI directs the synthesis of *N*-(butyryl)-l-homoserine lactone (C_4_-HSL) (de Kievit and Iglewski 2000; Rumbaugh et al. 2000). The two systems control the production of numerous virulence factors including LasB, LasA, rhamnolipids, alkaline protease, exotoxin A, pyocyanin, hydrogen cyanide, and the cytotoxic lectin LecA (Winson et al. 1995; de Kievit and Iglewski 2000; Rumbaugh et al. 2000; Yoon et al. 2002; Hentzer et al. 2003; Schuster et al. 2003; Wagner et al. 2003; Schuster and Greenberg 2006). Besides the *las* and the *rhl* systems, *P. aeruginosa* contains a third QS system that functions through the 2-alkyl-4-quinolone (AQ) signaling molecules (Lepine et al. 2004). Although *P. aeruginosa* produces numerous AQs, the two main AQs that function as QS signals are 2-heptyl-3-hydroxy-4-quinonlone (*Pseudomonas* quinolone signal, PQS) and its precursor 2-heptyl-4-quinolone (HHQ) (Pesci et al. 1999; Deziel et al. 2004; Diggle et al. 2007). Both AQs activate MvfR/PqsR, which enhances expression of the biosynthetic genes *pqsA*-*E* that synthesize HHQ (Xiao et al. 2006; Diggle et al. 2007). Previous studies identified several positive and negative regulators that regulate the *P. aeruginosa* QS systems including, Vfr, VqsR, MvaT, GacA, RpoN, RpoS, and RsmA (Albus et al. 1997; Reimmann et al. 1997; Chugani et al. 2001; Pessi et al. 2001; Diggle et al. 2002; Heurlier et al. 2003; Juhas et al. 2004).

Several previous studies demonstrated the influence of QS systems on the pathogenesis of *P. aeruginosa*. For example, multiple studies strongly suggested that the *P. aeruginosa* QS systems within the lung of chronically infected CF patients are fully functional (Storey et al. 1997, 1998; Wu et al. 2000; Erickson et al. 2002). Analysis of sputum samples from CF patients indicated the presence of either 3OC_12_-HSL or C_4_-HSL, or both (Geisenberger et al. 2000; Singh et al. 2000). Additionally, transcriptional analysis of *P. aeruginosa* strains from the sputum samples of CF patients showed the presence of *lasI* and *rhlI* transcripts in these samples (Storey et al. 1998; Erickson et al. 2002). The level of *lasI* and *rhlI* transcripts correlated well with the level of *lasB*, *lasA*, and *toxA* transcripts (Storey et al. 1998; Erickson et al. 2002). Using different animal models, other studies demonstrated the relevance of the QS systems to the virulence of *P. aeruginosa* (Rumbaugh et al. 1999; Pearson et al. 2000; Wu et al. 2000). Using the thermally injured mouse model and the mouse models of acute and chronic lung infections, investigators compared the virulence of *P. aeruginosa* mutants defective in QS systems with that of their parent strains (Rumbaugh et al. 1999; Pearson et al. 2000). The mortality rate among thermally injured mice infected with *P. aeruginosa* defective in either the *las*, *rhl*, or both was significantly lower than in mice infected with the wild-type strain (Rumbaugh et al. 1999). Similarly, the mortality rate, as well as lung damage, produced in mice infected with QS mutants was significantly lower than those infected with the wild-type strain (Pearson et al. 2000). The role of the AQ-related system in *P. aeruginosa* virulence was also demonstrated in multiple studies. Using the thermally injured mouse model and the *Arabidopsis* leaf infiltration assay, Cao et al. (2001) showed that the virulence of a *P. aeruginosa* mutant defective in *mvfR/pqsR* was significantly lower than its parent strain. Besides functioning as a signal molecule, PQS produces an oxidative stress response (Haussler and Becker 2008), plays a role in biofilm development (Allesen-Holm et al. 2006), and chelates iron (Bredenbruch et al. 2006; Diggle et al. 2007).

Interestingly, by constructing a PAO1 isogenic mutant defective in *lasR, lasI, rhlR,* and *rhlI* genes, Lazenby et al. (2013) recently produced evidence indicating that the loss of the *las/rhl* systems does not completely compromise the virulence of *P. aeruginosa*. Compared with its parent strain, the mutant produced reduced levels of elastase and proteases (Lazenby et al. 2013). However, in the mouse model of lung infection, the mutant was not defective; its persistence within the lung was equivalent to that of its parent strain (Lazenby et al. 2013). Additionally, the neutrophil infiltration as well as the expression of inflammatory cytokines within the lung was not diminished (Lazenby et al. 2013).

We previously showed that by enhancing twitching motility, serum interfered with biofilm development by *P. aeruginosa* (Hammond et al. 2010). Additionally, using transcriptome analysis, we recently investigated the effect of serum on the expression of different *P. aeruginosa* genes (Kruczek et al. 2012). At early stages of growth, serum repressed the expression of numerous *P. aeruginosa* iron-controlled genes (Kruczek et al. 2012). In this study, we examined the effect of serum on the expression of *P. aeruginosa* QS and QS-controlled genes.

## Experimental Procedures

### Bacterial strains, plasmids, media, and growth conditions

Bacterial strains and plasmids used in this study are listed in Table 1. Overnight cultures were grown in Luria Bertani (LB) broth at 37°C with shaking at 230 rpm. Aliquots of overnight cultures were pelleted, washed, and resuspended to a final OD_600_ of 0.02–0.03. Cultures were grown in LB or LB supplemented with 10% (v/v) adult bovine serum (LB/S) under conditions described above and samples taken at the indicated time points. We established a growth index of OD_600_ 1.0–1.2 achieved approximately 6-h postinoculation, as early exponential phase of growth (early phase) and a growth index of OD_600_ 3.0–4.0 achieved approximately 16-h postinoculation as late stationary phase of growth (late phase).

**Table 1:** Strains and plasmids utilized in this study.

Strains and plasmids	Description	Reference
*Pseudomonas aeruginosa*		
PAO1	Prototrophic PAO1	Holloway et al. (1979)
PAO1*lecA::lux*	*lecA:luxCDABE* genomic reporter fusion in PAO1; Tc^r^	Winzer et al. (2000)
PAOΔ*vfr*	*vfr* deletion of PAO1; Gm^r^	Runyen-Janecky et al. (1997)
PAOΔ*mvaT*	*mvaT* isogenic mutant of PAO1	Westfall et al. (2004)
PAO1*pqsA* CTX-*lux::pqsA*	*pqsA* mutant containing a copy of the *pqsA* promoter linked to the *luxCDABE* genes and inserted into a neutral site in the chromosome	Fletcher et al. (2007)
PW5352	*vqsR-*F12::IS*phoA*/hah in PAO1; out of frame; Tc^r^	Jacobs et al. (2003)
Plasmids		
pPCS223	*lasI-lasZ* transcriptional fusion in pLP170; Cb^r^	Preston et al. (1997)
pSB1142	AHL (C12) reporter plasmid; Tc^r^	Wang et al. (2007)
pSB536	AHL (C4) biosensor; *ahyR''*::*luxCDABE* in pAHP13; Cb^r^	Swift et al. (1997)

Gm, gentamicin; Tc, tetracycline; Cb, carbenicillin; ^r^, resistant.

### Assay for gene expression by β-galactosidase activity

Assays for *β*-galactosidase activity were performed as previously described (Miller 1972; Stachel et al. 1985; Gaines et al. 2005). The calculation for units of *β*-galactosidase activity takes into account the amount of growth. Results represent the averages of three independent experiments ± SEM.

### Detection of 3OC_12_-HSL, C_4_-HSL, and HHQ/PQS

*Pseudomonas aeruginosa* PAO1 was grown in triplicate in LB or LB/S until the indicated time points. The supernatant fraction was collected and passed through a 0.2-*μ*m syringe filter (VWR, Arlington Heights, IL). Autoinducers were separated from the supernatant via three successive rounds of acidified ethyl acetate (high performance liquid chromatography [HPLC] grade) extraction (Pearson et al. 1994). The final extracts were then dried in an Eppendorf 5301 concentrator (Eppendorf, Hauppauge, NY) and resuspended in 30 *μ*L of HPLC-grade methanol. HHQ is oxidized by the putative monooxygenase PqsH to produce PQS (Gallagher et al. 2002; Deziel et al. 2004). Therefore, the reporter strain detects both HHQ and PQS. Overnight cultures of the 3OC_12_-HSL, C_4_-HSL, and HHQ/PQS reporter strains (Table 1) were diluted to an OD_600_ of 0.5 in fresh LB. Aliquots (100 *μ*L) of the diluted reporter strains were pipetted into a 96-well clear flat-bottom microtiter plate (Costar, Corning, NY). Five-microliter aliquots of the methanol-dissolved autoinducer samples were then quickly added to the microtiter plate and the plate was incubated in the dark at 37°C for 3 h. Luminescence was detected using a luminometer (Modulus Microplate Reader, Turner Biosystems, Promega, Madison, WI). Values were standardized based on the OD_600_ value of each respective culture.

### Analysis of lecA expression

PAO1*lecA::lux* was grown in triplicate in LB or LB/S to the indicated time points and the OD_600_ values were obtained. A 100-*μ*L aliquot of each culture was added to a 96-well microtiter plate, and 20 *μ*L of 0.3% (v/v) decanal solution in water was then added to each well and quickly mixed with each culture. Luminescence was immediately measured using a luminometer (1 sec exposure). Reported values were standardized by dividing the relative light units (RLU) by the OD_600_ of the culture.

### RNA extraction and qRT-PCR analysis of gene expression

Overnight cultures of *P. aeruginosa* strains were subcultured in fresh LB or LB/S to an OD_600_ of 0.02 and incubated at 37°C with shaking to early and late phases of growth (˜6 and 16 h, respectively). Cultures were then mixed with twice of their volume of RNAprotect Bacteria Reagent (QIAGEN, Valencia, CA) for 5 min at room temperature. The cells were pelleted and were stored at −80°C. Bacterial pellets were first lysed with lysozyme (15 mg/mL) and proteinase K (1 mg/mL) for 15 min at room temperature and the RNA was subsequently extracted using the RNeasy Mini Kit (QIAGEN) according to the manufacturer's recommendations. The RNA solution was then digested with the RNase-free DNase Set (QIAGEN) to remove residual genomic DNA. RNA was purified from DNase by the RNA cleanup protocol (QIAGEN) with the exception that on-column DNase digestion was applied to eliminate any remaining traces of genomic DNA. RNA was quantified by NanoDrop® spectrophotometer (NanoDrop Products, Wilmington, DE) and the integrity of the RNA was assessed using RNA Nano Chip on an Agilent 2100 Bioanalyzer (Agilent, Santa Clara, CA).

Synthesis of cDNA from the extracted RNA was performed using the QuantiTect Reverse Transcription Kit (QIAGEN). A 200-ng aliquot of cDNA was mixed with SYBR Green PCR Master Mix (Life Technologies, Carlsbad, CA) and 250 nmol/L of specific primer (Table 2). Amplification and detection of the product were conducted using StepOne Plus real-time polymerase chain reaction (PCR) system (Life Technologies). For each experiment, we used three independent biological replicates for RNA extraction. Additionally, each PCR reaction was set up in triplicate. The quantity of cDNA in different samples was normalized using 30S ribosomal RNA (*rplS*) as an internal standard. Gene expression analysis was performed using StepOne Plus software version 2.2.2 (Life Technologies).

**Table 2:** Primers utilized in this study.

Gene	Forward primer	Reverse primer
*lasI*	5′-TTCCGACTGTACGCTGGAG-3′	5′-ATCTGGGTCTTGGCATTGAG-3′
*lasR*	5′-TTCTGGGAACCGTCCATCTA-3′	5′-CAGTGCGTAGTCCTTGAGCA-3′
*lasB*	5′-GTTCTATCCGCTGGTGTCG-3′	5′-GCCCTTGATGTCGTAGC-3′
*lasA*	5′-CGTTCCTCTTCGTCTTGCTG-3′	5′-GCTCCAGGTATTCGCTCTTG-3′
*rsaL*	5′-GAGAGAACACAGCCCCAAAA-3′	5′-GATTGGCTTATCCCGAAGC-3′
*rhlI*	5′-CTCTCTGAATCGCTGGAAGG-3′	5′-CGACGATGTAGCGGGTTT-3′
*rhlR*	5′-GTTGCATGATCGAGTTGCTG-3′	5′-TGGATGTTCTTGTGGTGGAA-3′
*rhlA*	5′-CTGAAAGCCAGCAACCATC-3′	5′-GGCGGTGGTGTATTCGTC-3′
*pqsA*	5′-CAATACACCTCGGGTTCCAC-3′	5′-TGCCATAGCCGAAGAACATC-3′
*phzC1*	5′-GACCCTGCCGGTCTATCG-3′	5′-CAGCATCGACAGCTCGTAGT-3′
*hcnB*	5′-CTGACGGAAGTGACGGTAGC-3′	5′-CAGGTGGATGTGCGGTTG-3′
*rsmA*	5′-TGACGGTACTGGGTGTCAAA-3′	5′-CTTTCTGGATGCGCTGGTAA-3′
*gacA*	5′-GGTCGTGGTAGTCACCGTCT-3′	5′-AAGGACTTCAGCGCCAGTT-3′
*vfr*	5′-TACCCACACACCCAAACTCA-3′	5′-GTTCGCTGCCTTCCTTTTC-3′
*vqsR*	5′-TTATGTGGTGACGGACGAAG-3′	5′-CTCGAAGTGGATGCGTTTTT-3′
*qscR*	5′-TCAAGAATAACAACCGAGGAAGA-3′	5′-CGGGTCGATGGATGTGTAGT-3′
*rpoN*	5′-AACGCATACCCAGCGAGTT-3′	5′-TCGAGGTAGCCATCGTTGTT-3′
*mvaT*	5′-ACGCTGATGGGCACTTAC-3′	5′-CACTTGGCTTTCCACTCTTT-3′

### Effect of serum on P. aeruginosa virulence factor production

Overnight cultures of *P. aeruginosa* PAO1 were subcultured in fresh LB or LB/S to an OD_600_ of 0.02 and incubated at 37°C with shaking to the early and late phases of growth. For the detection of elastolytic activity we utilized the elastin plate assay as described by Ohman et al. (1980). Elastin plates prepared with and without 10% serum were incubated for 24 h at 37°C and then 48 h at room temperature (Ohman et al. 1980). Staphylolytic activity was determined as previously described in Diggle et al. (2002). Phospholipase C activity was assayed as previously described using nitrophenylphosphoryl choline as a substrate (Berka et al. 1981; Barker et al. 2004).

### Statistical analyses

Statistical analyses were done using GraphPad InStat 3.06 (GraphPad Software, San Diego, CA). One-way analysis of variance (ANOVA) with the Tukey–Kramer multiple comparisons posttest was used to determine significant differences across time. The two-tailed *t*-test was used to compare pairs of strains grown in LB with those grown in LB/S.

## Results

### Serum differentially regulates the expression of PAO1 QS-related genes during growth

**Figure 1:**
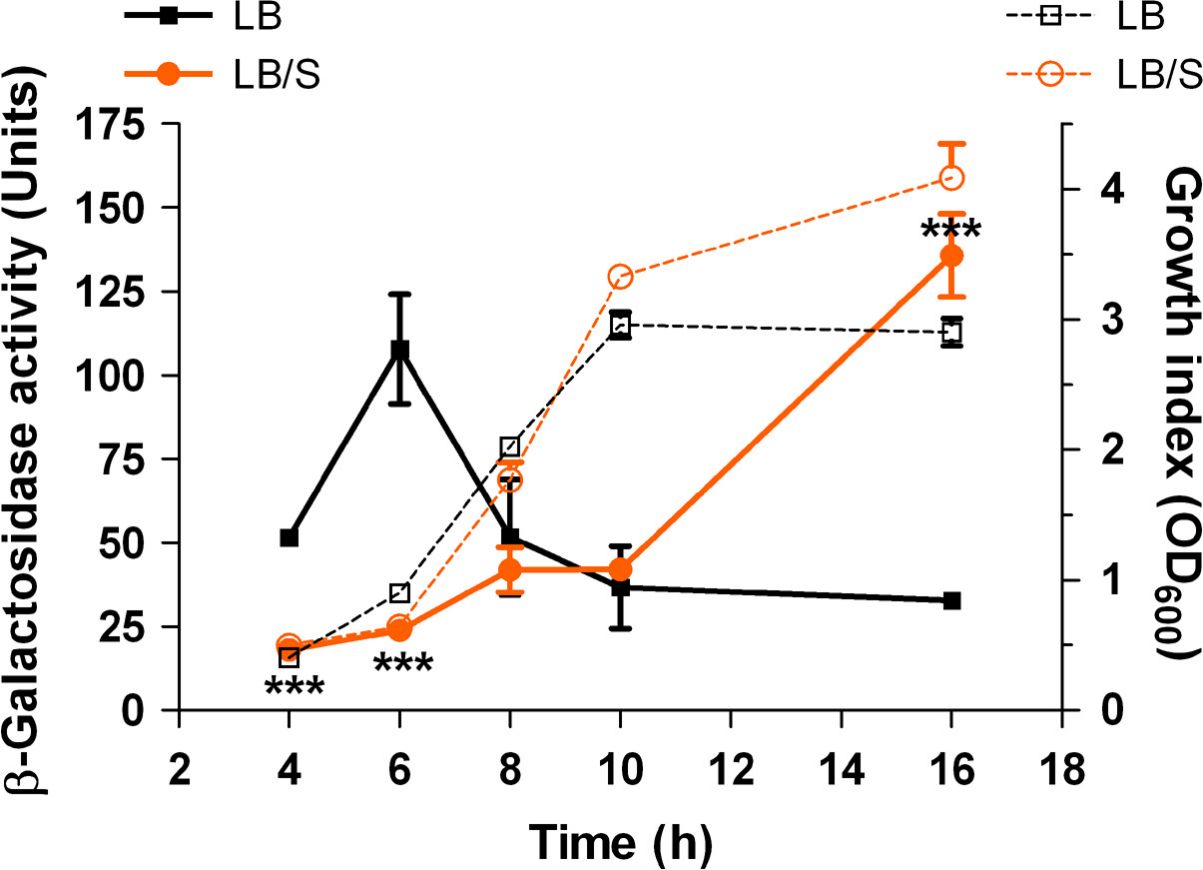
Serum represses *lasI* expression during early stages of growth, but enhances expression during late stages of growth. PAO1 carrying the *lasI*-*lacZ* fusion plasmid pPCS223 (Table 1) was grown in either LB or LB/S. Samples were obtained at the indicated time points, and the level of *β*-galactosidase activity and the growth index (OD_600_) were determined. Solid lines indicate units of *β*-galactosidase activity, whereas dashed lines indicate cell growth. Values represent the means of three independent experiments ± SEM; ***P < 0.001.

Through transcriptome analysis, we previously identified several PAO1 genes whose expression is significantly altered by serum. Most of these genes were iron-controlled genes. However, we also identified the QS gene *lasI* whose expression was repressed 20-fold by serum at early stages of growth. We confirmed the negative regulation of the *lasI* gene by serum using PAO1 carrying the *lasI-lacZ* transcriptional fusion plasmid pPCS223. To optimize QS gene expression, cells were grown in LB or LB/S for 16 h. Samples were obtained at the indicated time points throughout the growth cycle and the level of *β*-galactosidase activity was determined. During the early to midexponential phases growth (4–8 h, OD_600_ 0.8–1.8), *lasI* expression was reduced in the presence of serum (Fig. 1). However, at the late stationary phase of growth (16 h, OD_600_ 3.0–4.0), *lasI* expression was significantly enhanced by serum (Fig. 1). For the remainder of the study, we used a growth index of OD_600_ 1.0–1.2 (early phase) and a growth index of OD_600_ 3.0–4.0 (late phase) to provide consistency of results.

**Figure 2:**
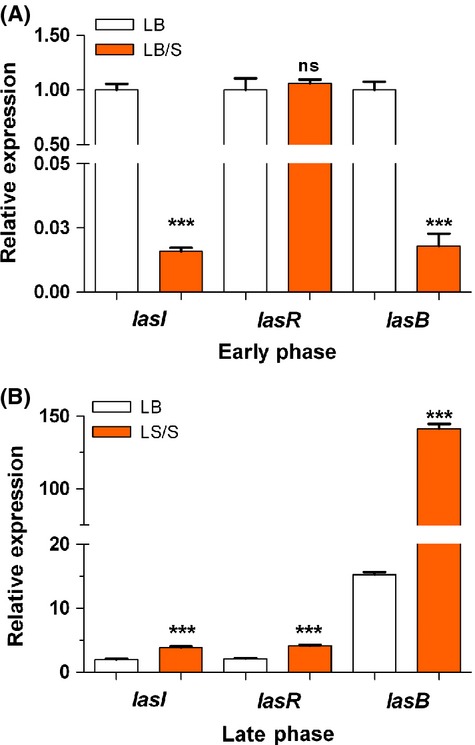
Serum represses *lasI* and *lasB* expression during early phase (A) but enhances *lasI*, *lasR*, and *lasB* expression during late phase (B) of growth. PAO1 was grown in either LB or LB/S. Samples were collected at early phase (OD_600_ 1.0–1.2) or late phase (OD_600_ 3.0–4.0) and the level of gene expression was determined by qRT-PCR. Values represent the means of three independent experiments ± SEM; ***P <* *0.001.

*Pseudomonas aeruginosa* has three QS systems – *las*, *rhl,* and the AQ-related system. Therefore, we decided to comprehensively analyze the effect of serum on the expression of different PAO1 QS genes within each system. To avoid problems associated with the presence of multiple copies of the regulated genes in a single cell, we examined the expression of the QS genes by qRT-PCR, rather than using *lacZ* fusion constructs carried on plasmids. We grew PAO1 in either LB or LB/S and obtained triplicate samples at early and late phases of growth and analyzed the expression of QS genes. To analyze the *las* system, we examined the level of expression of *lasR*, *lasI*, and *lasB*, a virulence gene that is stringently regulated by the *las* system. At early phase, the level of *lasI* and *lasB* expression in LB/S was significantly lower than that in LB, but the level of *lasR* expression was essentially unchanged (Fig. 2A). In contrast, at the late phase, the level of expression of all three genes was significantly enhanced in LB/S compared with LB (Fig. 2B).

**Figure 3:**
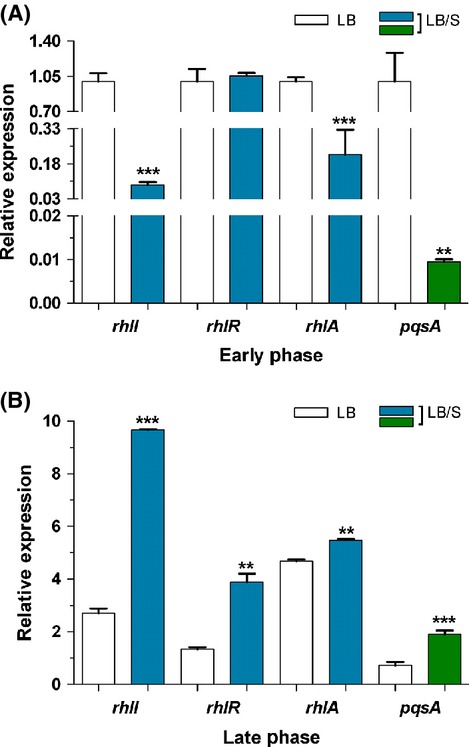
Serum represses *rhlI*, *rhlA*, and *pqsA* expression during early phase (A) but enhances *rhlI*, *rhlR*, *rhlA*, and *pqsA* expression during late phase (B) of growth. PAO1 was grown in either LB or LB/S. Samples were collected at early and late phases and the level of gene expression was determined by qRT-PCR. Values represent the means of three independent experiments ± SEM; ***P <* *0.001; **P < 0.01.

Using the same approach, we analyzed the effect of serum on the expression of the *rhl* genes, *rhlI*, *rhlR*, and *rhlA*. Similarly to its effects on the *las* genes, serum repressed *rhlI* and *rhlA* expression significantly in the early phase but enhanced their expression significantly in the late phase, although the enhancement in *rhlA* expression was not as pronounced as that seen with *lasB* (Fig. 3A and B). As we observed with *lasR* expression, serum had no significant effect on *rhlR* expression at early phase, but enhanced its expression at late phase (Fig. 3A and B). HHQ and PQS are synthesized by proteins encoded by the *pqsA-D* genes of the *pqsA-E* operon. Specifically, these proteins synthesize HHQ, which is then converted into PQS by the potential monooxygenase encoded by *pqsH* (Diggle et al. 2002; Deziel et al. 2004). The potential monooxygenase encoded by *pqsH* converts HHQ into PQS (Gallagher et al. 2002; Deziel et al. 2004). The *pqsA-E* operon is positively regulated by MvfR/PqsR (Cao et al. 2001; Gallagher et al. 2002). Therefore, we examined the expression of *pqsA* as a representative gene from the operon. Similar to the pattern of *lasI* and *rhlI* expression, serum significantly repressed *pqsA* expression at early phase, but significantly enhanced it at the late phase (Fig. 3C). These results suggest that, depending on the stage of growth, serum differentially regulates the expression of its target *las*, *rhl*, and *pqs* genes, repressing their expression at early stages of growth but enhancing it at late stages of growth. Additionally, the regulation at early stages of growth is limited to the *lasI* and *rhlI* genes.

We excluded the possibility that serum interacts with the autoinducers and either modifies or alters their function. We incubated the autoinducers with either LB or LB/S and reextracted them. The reextracted autoinducers maintained their ability to significantly enhance *lasB* expression in PAO1 (data not shown).

### Serum differentially regulates the production of PAO1 autoinducers 3OC_12_-HSL, C_4_-HSL, and HHQ/PQS at early and late phases of growth

**Figure 4:**
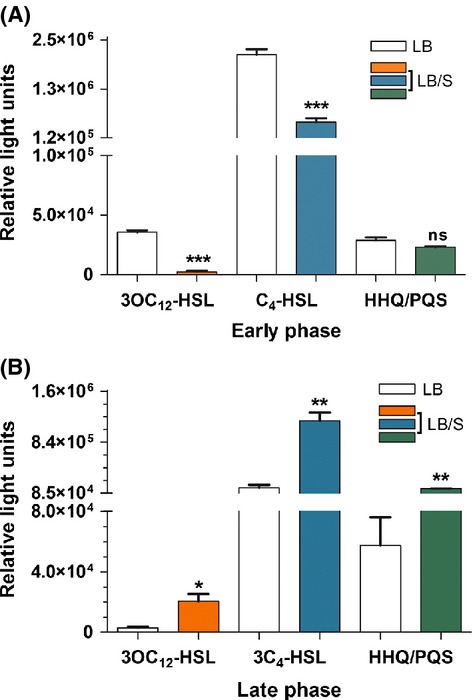
Serum represses 3OC_12_-HSL and C_4_-HSL production during early phases (A) but enhances 3OC_12_-HSL, C_4_-HSL, and HHQ/PQS production during late phases (B) of growth. PAO1 was grown in either LB or LB/S. Samples were collected at early and late phases, autoinducers were extracted from the supernatant and their levels were determined as described in Experimental Procedures. Values represent the means of three independent experiments ± SEM; ***P <* *0.001; **P < 0.01; *P <* *0.05; ns, not significant.

The *lasI*, *rhlI*, and *pqsA-E* genes encode synthases that produce the 3OC_12_-HSL, C_4_-HSL, and HHQ autoinducers, respectively. Therefore, we analyzed the effect of serum on the level of 3OC_12_-HSL, C_4_-HSL, and HHQ/PQS produced by PAO1. Cells were grown in either LB or LB/S to early and late phases of growth and the level of autoinducer was detected using their respective autoinducer assays as previously described (Swift et al. 1997; Winson et al. 1998; Diggle et al. 2002). At early phase and in the presence of serum, PAO1 produced significantly lower levels of 3OC_12_-HSL and C_4_-HSL than PAO1 grown in LB only (Fig. 4A). However, serum had no significant effect on HHQ/PQS production (Fig. 4A). At late phase, serum significantly enhanced the production of all three autoinducers (Fig. 4B). These results suggest that serum differentially regulates the production of the three autoinducers by significantly repressing 3OC_12_-HSL and C_4_-HSL production at earlier stages of growth but significantly enhancing them at late stages of growth.

We ruled out the possibility that serum contains certain factors or molecules that affect the level of the luminescence produced by the reporter strains. We prepared ethyl acetate extracts from either LB or LB/S. Compared with the negative control, to which no extract was added, the addition of ethyl acetate extract from LB or LB/S did not alter the level of the luminescence activity produced by the reporter strains (data not shown).

### Serum influences the expression of QS-controlled virulence genes

**Figure 5:**
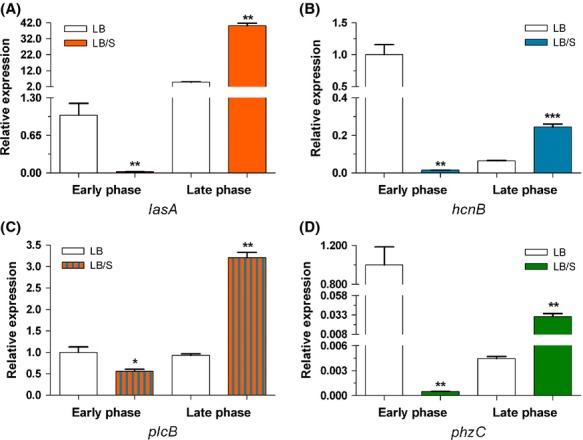
Serum differentially regulates the expression of the QS-controlled virulence genes (A) *lasA*, (B) *hcnB*, (C) *plcB*, and (D) *phzC* during both early and late stages of growth. PAO1 was grown in either LB or LB/S, samples were collected at early and late phases, and the level of gene expression was determined by qRT-PCR. Values represent the means of three independent experiments ± SEM; ***P <* *0.001, **P < 0.01, *P <* *0.05.

**Figure 6:**
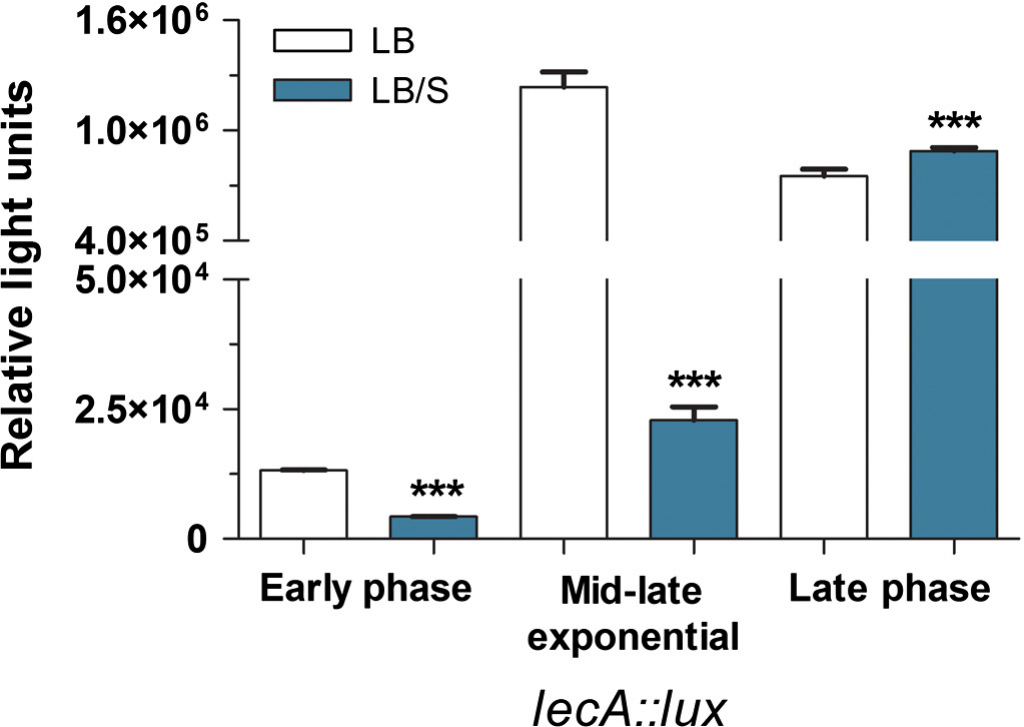
In the presence of serum, *lecA* expression is reduced at early phase but increased at late phase of growth of PAO1. PAO1*lecA::lux* was grown in LB or LB/S for 16 h. Samples were collected at early phase, midlate exponential phase (OD_600_ 2.0–2.2), and late phase. Relative luminescence values were determined as described in Experimental Procedures. Values represent the means of three independent experiments ± SEM; ***P <* *0.001.

The three QS systems control the expression of numerous virulence genes (de Kievit and Iglewski 2000; Rumbaugh et al. 2000; Smith and Iglewski 2003). In addition to the LasB elastase gene *lasB*, the expression of *lasA*, which encodes the staphylolytic protein LasA, is regulated by the *las* system (de Kievit and Iglewski 2000; Rumbaugh et al. 2000; Smith and Iglewski 2003). Besides *rhlAB*, expression of the *hcn* operon, which encodes proteins that direct production of hydrogen cyanide, is primarily regulated by the *rhl* system; whereas expression of *plcB*, which encodes the third *P. aeruginosa* phospholipase C protein PlcB, is controlled by both *las* and *rhl* (Reimmann et al. 1997; Barker et al. 2004). Pyocyanin production by *P. aeruginosa* is positively regulated by *pqsE* which is part of the *pqsA-E* operon (Farrow et al. 2008; Rampioni et al. 2010). Both HHQ and PQS bind to and activate MvfR/PqsR, which in turn binds to the *pqsA* promoter and enhances *pqsA-E* expression (McGrath et al. 2004; Wade et al. 2005; Xiao et al. 2006; Diggle et al. 2007). Therefore, HHQ and PQS indirectly regulate pyocyanin production by enhancing *pqsE* expression. To determine if serum affects additional QS-controlled virulence genes (besides *lasB* and *rhlA*), we analyzed the expression of *lasA*, *hcnB*, *plcB*, and *phzC* using qRT-PCR. The expression of *phzC* and *hcnB* served to represent the *phz* and *hcn* operons, respectively. As observed with *lasB* and *rhlA*, in the presence of serum, the expression of all four genes was significantly reduced in the early phase of growth and significantly enhanced in the late phase (Fig. 5). We also examined the effect of serum on the expression of *lecA* which encodes a cytotoxic lectin (Diggle et al. 2006). This gene is regulated by the QS-controlled sigma factor RpoS and the *rhl* QS system (Winzer et al. 2000). To determine the effect of serum on *lecA* expression, we utilized the *P. aeruginosa* strain PAO1*lecA::lux* which carries a *lecA::lux* chromosomal fusion (Winzer et al. 2000; Diggle et al. 2002). At the early phase of growth, *lecA* expression was relatively low (Fig. 6). Therefore, we grew PAO1*lecA::lux* for 8 h to an OD_600_ of 2.0–2.4, or mid-to-late exponential phase (midlate exponential phase). At both early and midlate exponential phases, serum significantly reduced the level of *lecA* expression (Fig. 6). In contrast, by late phase, serum significantly enhanced *lecA* expression (Fig. 6). These results suggest that in response to the effect of serum and depending on the stage of growth of PAO1 QS-controlled genes are differentially expressed in the same manner as their cognate QS genes.

### QS-controlled virulence factor production is altered in the presence of serum

**Figure 7:**
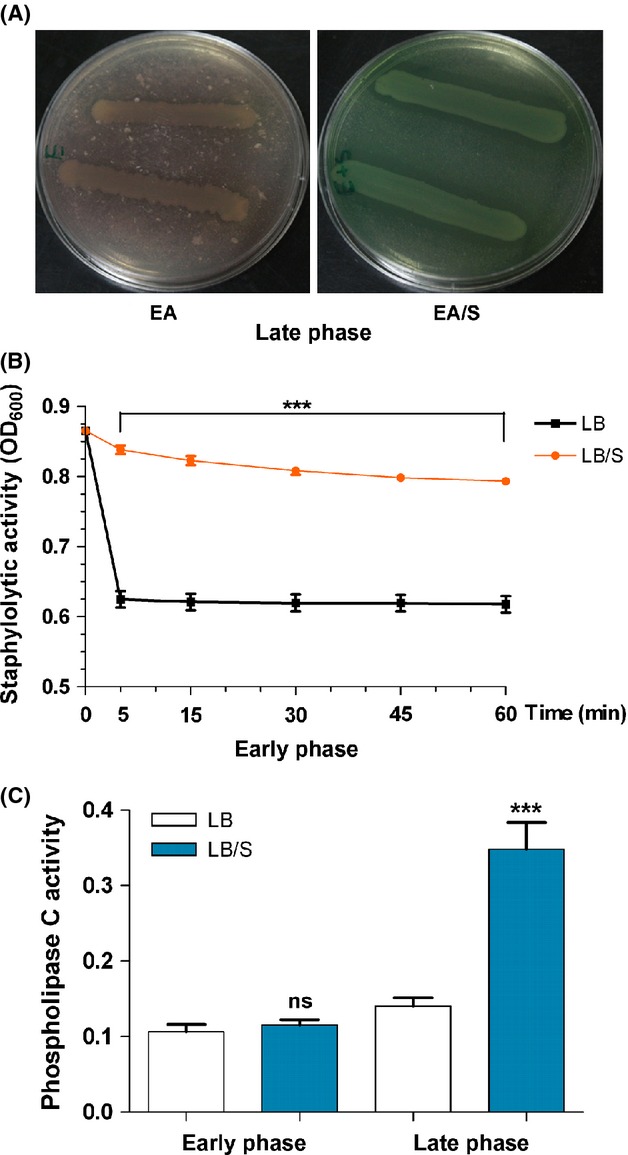
Serum influences the production of the PAO1 QS-controlled virulence factors, LasB, LasA, and phospholipase C. (A) At late phase of growth, serum enhances LasB production. PAO1 was streaked on elastin agar (EA) or elastin agar with 10% serum (EA/S). The plates were incubated at 37°C for 24 h and then at room temperature for 48 h before examination. Images are representative of three independent experiments. (B) Serum represses LasA production at early phase. PAO1 was grown in either LB or LB/S and samples were collected at the early phase. The supernatant fractions were isolated and the level of LasA activity in each fraction was determined using the staphylolytic assay as described in Experimental Procedures. (C) In the presence of serum, PAO1 produced increased levels of phospholipase C activity at late phase. Cells were grown as described in (B). Samples were collected at early and late phases, the supernatant fractions were isolated, and the level of phospholipase C activity in each fraction was determined as described in Experimental Procedures. For (B) and (C), values represent the means of three independent experiments ± SEM; ***P <* *0.001.

Using standard assays, we examined the effects of serum on the production of QS-controlled *P. aeruginosa* virulence factors. However, different components of serum interfered with many of these assays. Despite that, we obtained results that support our above described transcriptional studies at either early or late stages of growth. For example, we attempted to analyze *lasB* activity using the elastin Congo red assay, but serum contains strong endogenous elastolytic activity that interfered with our analysis (data not shown). We were also unsuccessful in determining the level of LasB protein in the presence and absence of serum using either enzyme-linked immunosorbent assay (ELISA) or immunoblotting as serum contains high-molecular-weight proteins (80–150 kDa) that strongly interacted with the LasB antibody (data not shown). In a last attempt, we compared the elastolytic activity produced by PAO1 in the presence and absence of serum using the previously described elastin plate assay (Ohman et al. 1980). After 24 h of incubation at 37°C and 48 h at room temperature, PAO1 colonies on the serum-containing elastin plates produced a zone of elastin proteolysis that was larger than that produced around PAO1 colonies on a regular elastin plate (Fig. 7A). Although these results support the assumption that serum affects LasB production through QS genes at late stages of growth of PAO1, it does not address the potential reduction in LasB production at early stages of growth. Serum also interfered with the detection of pyocyanin within the supernatant of PAO1. However, we were able to examine the effect of serum on LasA production using the previously described staphylolytic assay (Diggle et al. 2002). In the presence of serum and at early phase, PAO1 staphylolytic activity was significantly reduced (Fig. 7B). Using the phospholipase C assay (Berka et al. 1981), we determined the level of phospholipase C activity within the supernatant of PAO1 in the presence and absence of serum. At late phase, serum significantly enhanced phospholipase C production (Fig. 7C).

### Serum influences the expression of PAO1 genes that regulate the QS systems

**Figure 8:**
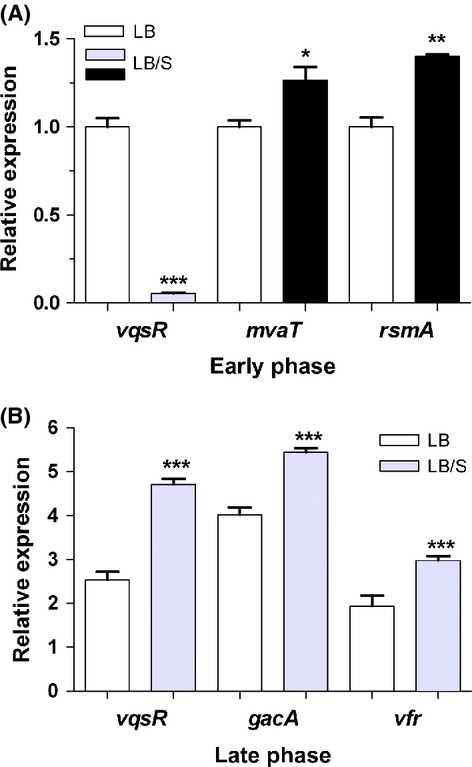
Serum differentially regulates the expression of the QS regulatory genes during early and late phases. PAO1 was grown in either LB or LB/S. Samples were obtained at early and late phases and the level of expression was determined by qRT-PCR analysis. (A) At early phase, serum reduced the expression of the PAO1 QS-positive regulator *vqsR*, but enhanced the expression of the QS-negative regulators *mvaT* and *rsmA*. (B) At late phase, serum enhanced the expression of the QS-positive regulators *vqsR*, *gacA*, and *vfr*. Values represent the means of three independent experiments ± SEM; ***P <* *0.001, **P < 0.01, *P <* *0.05.

Previous studies identified and characterized numerous *P. aeruginosa* genes that positively or negatively regulate the *las*, *rhl*, and the AQ-related QS systems. Among the positive regulators are *vfr*, *gacA*, and *vqsR* and among the negative regulators are *mvaT*, *rsmA*, *qscR*, *rpoS*, and *rpoN* (Albus et al. 1997; Reimmann et al. 1997; Chugani et al. 2001; Pessi et al. 2001; Diggle et al. 2002; Heurlier et al. 2003; Juhas et al. 2004, 2005). Therefore, we determined if serum affected the expression of any of the QS genes (*las*, *rhl*, and PQS) through one or more of these regulators. At early phase, serum significantly reduced expression of the positive regulator *vqsR*, but enhanced the expression of the negative regulators *mvaT* and *rsmA* (Fig. 8A). Serum produced no significant effect on the expression of *gacA*, *rpoN*, *qscR*, or *rpoS* at early phase (data not shown). In the late phase, serum significantly enhanced the expression of the QS-positive regulators *vqsR*, *gacA*, and *vfr* (Fig. 8B). While serum minimally reduced expression of the negative regulators *rpoN* and *qscR*, this reduction was not significant (data not shown). Again, there was no significant effect on the expression of *mvaT*, *rsmA*, or *rpoS* (data not shown). These results suggest that at either early or late stages of growth, serum affects the expression of one or more of the QS gene regulators.

### At late stages of growth of PAO1 serum enhances *lasI* and *lasR* expression through *vfr*

**Figure 9:**
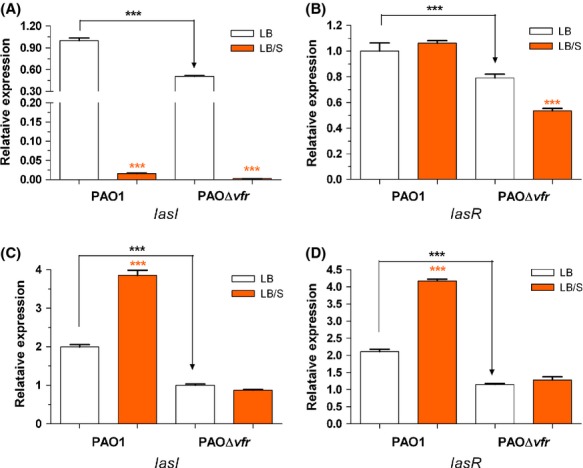
Deletion within *vfr* eliminated the effect of serum on *lasI*/*lasR* expression at late but not early phase. PAO1 and PAOΔ*vfr* were grown in either LB or LB/S to either early phase (A and C) or late phase (B and D). Samples were collected at each phase and the level of *lasI* (A and C) and *lasR* (B and D) expression was determined by qRT-PCR analysis. Values represent the means of three independent experiments ± SEM; ***P <* *0.001.

To determine if serum produces its effect on the *las/rhl* genes through *vfr*, we compared the level of *lasI*, *lasR*, *rhlI*, and *rhlR* expression between PAO1 and the *vfr* deletion mutant PAOΔ*vfr* at early and late phases of growth. At early phase and in LB, the level of expression of all these genes in PAOΔ*vfr* was significantly lower than that in PAO1 confirming the importance of *vfr* in regulating the QS genes (Fig. 9). Serum significantly reduced *lasI* expression in PAO1 and PAOΔ*vfr* at early phase (Fig. 9A). As we previously observed, serum did not affect *lasR* expression in PAO1 at early phase, however, its presence significantly repressed the expression of *lasR* in PAOΔ*vfr* (Fig. 9B). This suggests that at early stages of growth serum does not repress *lasI* expression through *vfr*, which supports our above finding regarding *vfr* regulation by serum in the early phase of growth. Additionally, at this stage of growth, intact *vfr* may be required to prevent the repression of *lasR* expression by serum (Fig. 9B). At late phase and in LB, the expression of *lasI* and *lasR* is reduced in PAOΔ*vfr* compared with PAO1 (Fig. 9C and D). However, serum enhancement of *lasI* and *lasR* expression was abrogated in PAOΔ*vfr* (Fig. 9C and D). These results suggest that in the late phase of growth, serum enhances the expression of *las* genes through *vfr*.

### At early stages of growth serum does not enhance *lasI* expression through *vqsR* or *mvaT*

**Figure 10:**
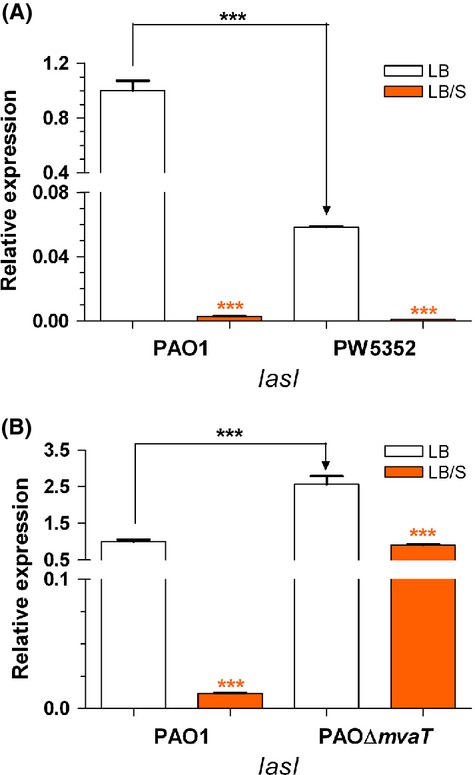
A mutation in either *vqsR* or *mvaT* does not interfere with the ability of serum to repress *lasI* expression during early phase of growth. (A) PAO1 and PW5352 (*vqsR*::*phoA*) were grown in either LB or LB/S to early phase. (B) PAO1 and PAOΔ*mvaT* were grown in either LB or LB/S to early phase. The level of *lasI* and *lasR* expression was determined by qRT-PCR analysis. Values represent the means of three independent experiments ± SEM; ***P <* *0.001.

Juhas et al. (2004) previously suggested that VqsR positively regulates *lasI* expression and AHL synthesis. As we demonstrated in Figure 9, serum significantly reduced *vqsR* at the early phase of growth of PAO1. To determine if serum potentially regulates *lasI/lasR* expression at this stage through VqsR, we utilized the *vqsR* transposon insertion mutant PW5352 (Table 1). In LB and compared with PAO1, the expression of *lasI* in PW5352 was significantly reduced indicating that VqsR is a positive regulator of the QS systems (Fig. 10A). However, in LB/S the expression of *lasI* was significantly reduced in both PAO1 and PW5352 indicating that serum represses the expression of these genes independently of VqsR (Fig. 10A). MvaT, which belongs to the family of histone-like nucleoid-structuring proteins, controls the timing of QS-dependent gene expression (Diggle et al. 2002; Tendeng et al. 2003; Vallet et al. 2004). Mutations in the *mvaT* gene cause a premature expression of the QS genes (Diggle et al. 2002). We showed that serum enhances *mvaT* expression at the early phase of growth (Fig. 8A). Therefore, to determine if serum reduces the expression of *lasI* at this stage of growth through *mvaT*, we compared the level of *lasI* expression between PAO1 and its *mvaT* mutant. In agreement with the results of previous studies, in LB the level of *lasI* expression in PAOΔ*mvaT* was significantly higher than that in PAO1 (Fig. 10B). However, in the presence of serum, the level of *lasI* expression was significantly reduced in PAO1 and PAOΔ*mvaT* suggesting that serum does not repress *lasI* expression through *mvaT* (Fig. 10B).

## Discussion

In this study, we demonstrated that depending on the stage of growth of *P. aeruginosa*, serum differentially regulates the expression of different QS-controlled genes. In the early phase of growth, serum repressed the expression of *lasI*, *lasB*, *rhlI*, *rhlA*, and *pqsA* (Figs. 2A, 3A). At the late phase, serum enhanced the expression of *lasI*, *lasR*, *lasB*, *rhlI*, *rhlR*, *rhlA*, and *pqsA* (Figs. 2B, 3B and C). Similarly to its effect on the QS systems, serum reduces the production of 3OC_12_-HSL and C_4_-HSL autoinducers at the early phase and enhances the production of 3OC_12_-HSL, C_4_-HSL, and HHQ/PQS at late phase of growth (Fig. 4). In the early phase of growth, serum does not accomplish its effect through any of the known QS regulators. However, in the late phase, serum utilizes *vfr* to influence the QS systems (Fig. 9).

With respect to the *las* and *rhl* QS systems and at early phase, the serum-induced reduction in the expression of QS-controlled virulence genes occurs by reducing the autoinducer synthases but not the transcriptional activators (Figs. 2A, 3A). Expression of *lasI* and 3OC_12_-HSL production were reduced but not *lasR* transcription (Figs. 2A, 4A). Similarly, the reduction involves *rhlI* transcription and C_4_-HSL production but not *rhlR* transcription (Figs. 3A, 4A). Synthesis of LasR and RhlR is regulated by the growth conditions that affect the metabolic activity of *P. aeruginosa* as well as the growth phase (Pesci et al. 1997; Medina et al. 2003). Such regulation is accomplished through several previously identified regulators. For example, RpoS, MvaT, and QscR regulate the timing of activation of the QS genes; rather than repressing the activated QS systems, these regulators prevent their premature activation (Whiteley et al. 2000; Chugani et al. 2001; Diggle et al. 2002). However, no previous study described either growth or an environmental condition that represses the expression of the already activated QS system. In this study, serum does not interfere with the timing of activation of the QS genes. Rather, serum represses the expression of *lasI* and *rhlI* genes at the same time when both genes are maximally expressed in PAO1 grown in LB only (Fig. 1, data not shown). In this regard, serum basically shuts down the QS systems at the time of their maximum function. This effect may be related to the interaction of different components of the QS system in response to an environmental stimulus as the growth phase changes. Upon its multimerization by 3OC_12_-HSL, LasR activates the transcription of its target genes (Kiratisin et al. 2002). RhlR, on the other hand, requires C_4_-HSL for its activation but does not require it for dimerization (Pearson et al. 1995). Additionally, through a positive feedback system, both LasR and RhlR activate the transcription of *lasI* and *rhlI*, respectively, thereby amplifying the activation signal (Latifi et al. 1996; Pesci et al. 1997). The autoinducers HHQ/PQS activate the LysR transcriptional activator MvfR/PqsR, which enhances the transcription of the *pqsA*-*E* operon (McGrath et al. 2004; Wade et al. 2005; Xiao et al. 2006). As *P. aeruginosa* reaches quorum, the initial response involves an increase in *lasI* and *rhlI* transcription which leads to an increased production of 3OC_12_-HSL and C_4_-HSL. At this stage, both LasR and RhlR are only activated by their respective autoinducers. This activation at this stage may not involve an increase in either *lasR* or *rhlR* transcription.

**Figure 11:**
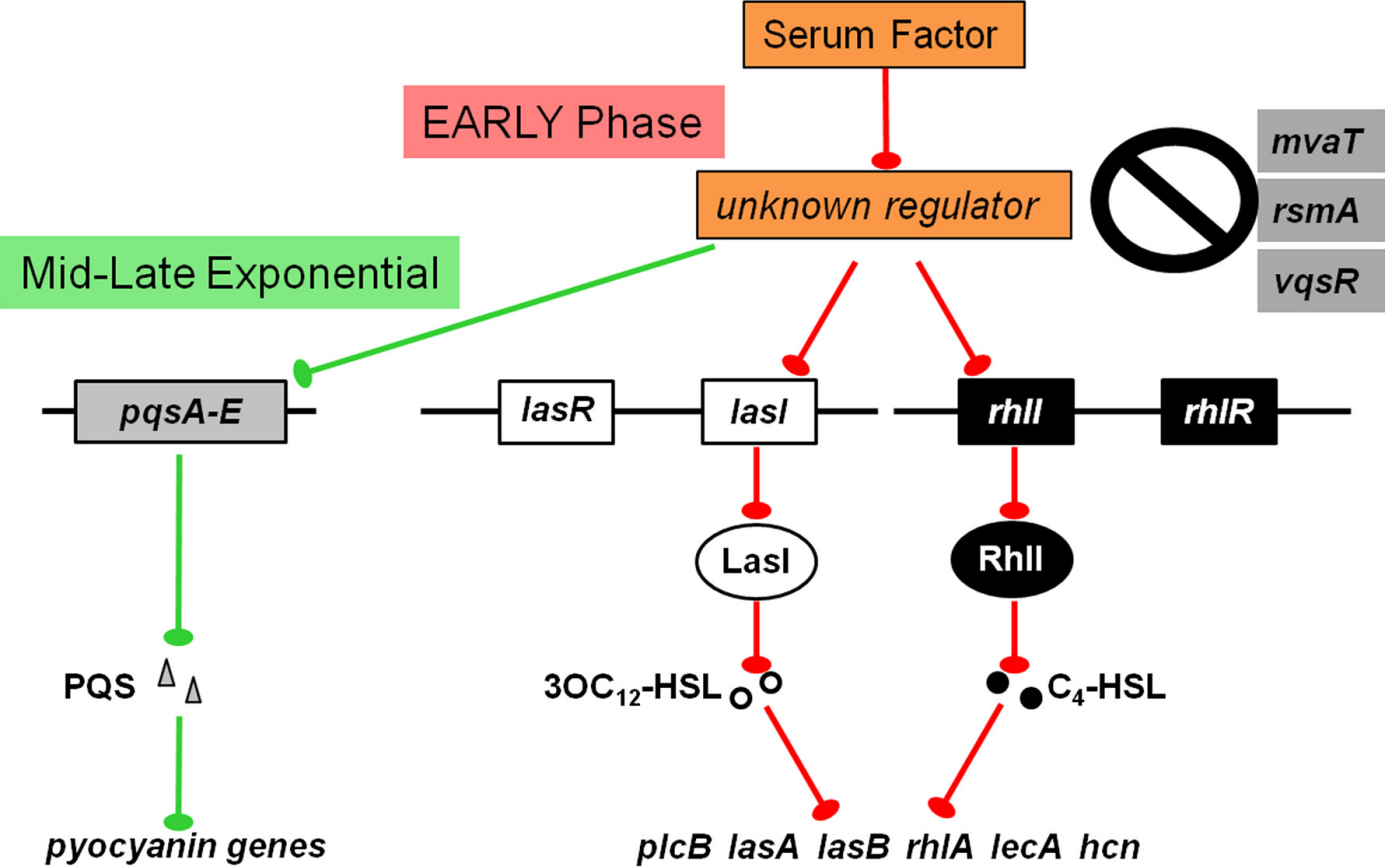
Diagram illustrating the possible mechanism through which serum represses the expression of PAO1 QS and QS-controlled virulence genes. The specific gene(s) through which serum regulates the QS systems is not known at this time. Blunted arrows indicate repression.

One possible scenario to explain the observed effect of serum at early growth phases is that at quorum and rather than reducing the expression of QS genes, serum interferes with *lasI* and *rhlI* activation (Fig. 11). Consequently, the amount of synthesized 3OC_12_-HSL and C_4_-HSL is not sufficient to activate LasR and RhlR and no increase in the transcription of the QS-controlled genes occurs. In contrast, in LB and at quorum, induction of *lasI* and *rhlI* expression produces sufficient amounts of 3OC_12_-HSL and C_4_-HSL to fully activate LasR and RhlR. Activated LasR and RhlR in turn produce maximum expression of the QS-controlled virulence genes. Our results suggest that the effect of serum is direct. Serum is less likely to interfere with the QS systems by binding the autoinducer extracellularly and reducing their availability to activate LasR and RhlR. Rather, serum appears to prevent LasI and RhlI activation and reduces the amount of the synthase required to produce the autoinducers (Fig. 11).

To determine the mechanism by which serum affects QS gene expression at early phases of growth, we sought to identify the gene(s) through which this may occur. We compared the expression of several QS regulatory genes in the presence and absence of serum. Although the expression of many of these genes was not affected, the expression of *vqsR*, *rsmA*, and *mvaT* was affected (Fig. 8). The QS genes are positively regulated by *vqsR* and negatively regulated by *rsmA* and *mvaT* (Pessi et al. 2001; Diggle et al. 2002; Juhas et al. 2004). To determine if serum regulation of QS systems occurs through any of these genes, we compared the level of *lasI* expression between PAO1 and PAO1 mutants defective in *mvaT* and *vqsR* in LB and LB/S. Serum still repressed *lasI* expression in a PAO1 mutant defective in *mvaT* as well as a PAO1 mutant defective in *vqsR* suggesting that neither gene is the potential candidate (Fig. 10). Therefore, the potential gene through which serum regulates the QS systems is not known at this time (Fig. 11).

**Figure 12:**
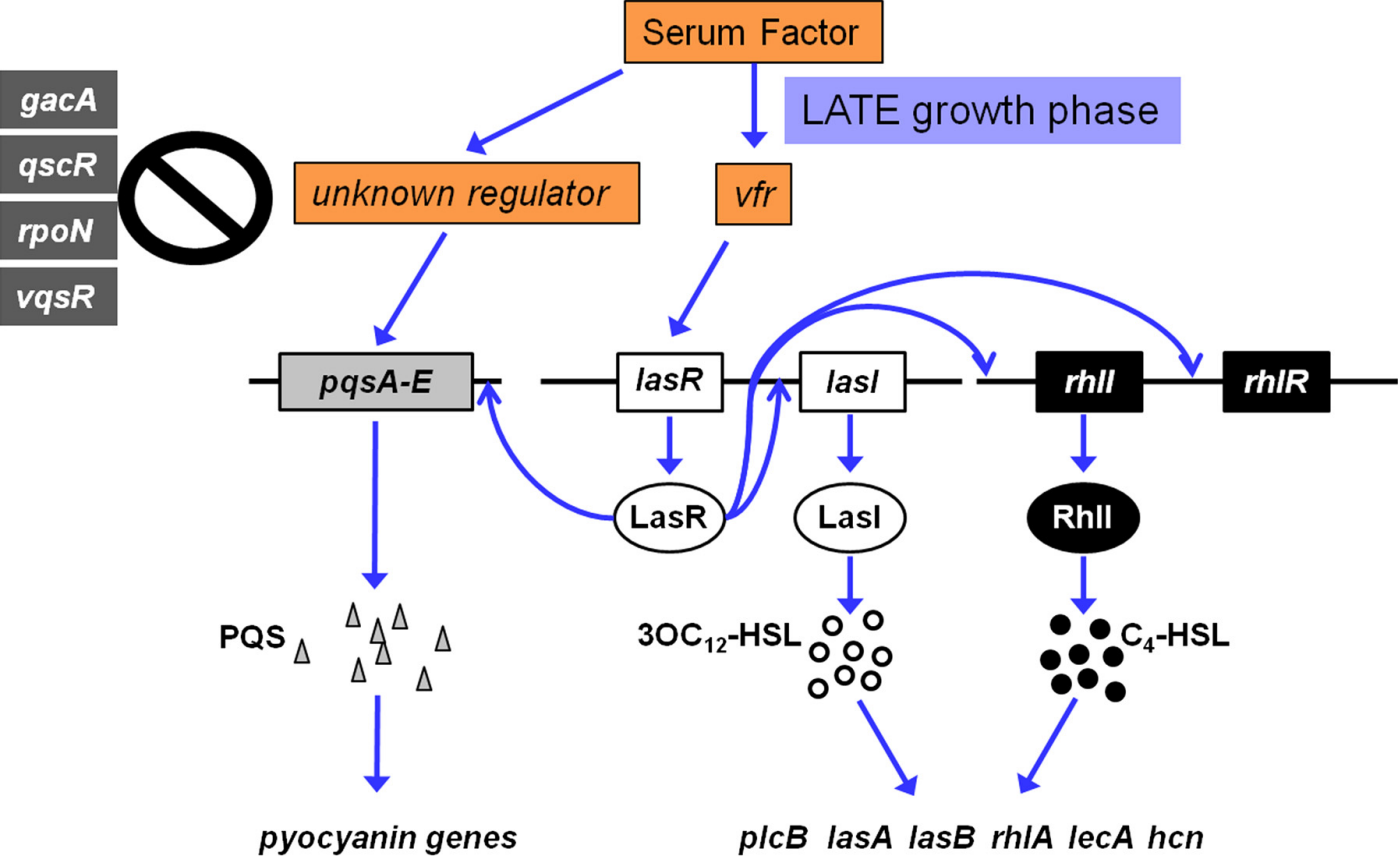
Diagram illustrating the possible mechanism through which serum enhances the expression of PAO1 QS and QS-controlled virulence genes. Our results suggest that serum regulates the QS genes through *vfr*. Serum enhances *vfr* expression, which increases the level of Vfr. Consequently, *lasR* expression is increased and LasR is activated, which enhances *lasI* transcription and *rhlI* and *rhlR* transcription is activated. Additionally, activated LasR induces *pqsA-E* expression.

At late stationary phases of growth, serum enhances the expression of the QS genes as well as QS-controlled virulence genes through *vfr*. At this stage of growth, serum increases the transcription of the QS-positive regulators *gacA*, *vqsR*, and *vfr* but reduced the transcription of the QS-negative regulator *rpoN* (Fig. 8, data not shown). Among the PAO1 mutants defective in each of these genes, only *vfr* deletion strains showed no change in *lasR* or *lasI* expression in response to serum (Fig. 9C and D). All or most of the serum effect is likely to occur through *vfr*. At late stationary phase of growth and compared with PAO1, the enhancement in *lasR* and *lasI* expression in PAOΔ*vfr* by serum was abrogated (Fig. 9C and D). Vfr, which is a member of the cyclic cAMP receptor protein family of transcriptional regulators, positively regulates the expression of the *lasR* and *rhlR* genes and numerous QS-dependent and QS-independent *P. aeruginosa* virulence genes (West et al. 1994; Albus et al. 1997). The flagellar genes only are negatively regulated by Vfr (Vfr represses the expression of *fleQ*, which controls the expression of many flagellar genes) (Dasgupta et al. 2002). Vfr regulates its target genes by specifically binding to a *vfr* consensus sequence within the promoter region of these genes including *lasR* (Kanack et al. 2006). Upon its activation by cyclic AMP (cAMP), Vfr binds to most of these promoters (West et al. 1994; Suh et al. 2002). No Vfr binding has been demonstrated to either *lasI* or *rhlI* (Albus et al. 1997; Kanack et al. 2006). Therefore, the likely scenario to explain how serum affects the QS genes at late phase is that serum enhances *vfr* expression which increases the level of *Vfr* protein (Fig. 12). Consequently, the *lasR* gene is activated which enhances *lasI* transcription and increases 3OC_12_-HSL production (Fig. 12). Additionally, LasR activates *rhlR*/*rhlI* transcription, increasing C_4_-HSL levels. Furthermore, LasR enhances *mvfR* expression, inducing the transcription of the *pqsA*-*E* operon leading to increased levels of HHQ and PQS. Activation of these three systems would increase the transcription of different QS-controlled genes. It is important to note that in contrast to its effect at early growth phases, which is LasR independent, serum appears to accomplish its effects at late stationary phase mainly through LasR.

The serum-induced growth phase-dependent regulation of *P. aeruginosa* QS-controlled virulence factors has been previously described. We previously demonstrated that serum induces the expression of numerous PAO1 iron-regulated genes at early stages of growth (OD_600_ 0.7–1) (Kruczek et al. 2012). Serum had no effect on the expression of these genes at later stages of growth (Kruczek et al. 2012). Further analysis revealed that serum albumin induced the expression of these iron-regulated genes (Kruczek et al. 2012). Serum is a complex medium that contains proteins and factors that may positively or negatively regulate the expression of QS and QS-controlled genes. Depending on the efficiency of this regulation by each factor, the net regulation may either be positive or negative. These potential factors exist in the LB/S throughout the growth cycle. Therefore, the PAO1 response to serum at early or late stages of growth is likely dictated by certain metabolites or structural products that are induced at that specific stage of growth.

With respect to the enhancement in QS and QS-controlled virulence genes by serum, several previous studies support this observation. Juhas et al. (2004) previously showed that PAO1 QS and QS-controlled genes were among the 113 genes whose expression is enhanced by serum. Such an effect was eliminated by either *rhlI* or *rhlR* mutation (Juhas et al. 2004). Additionally, Wu et al. (2005) suggested that a specific binding of interferon gamma to the *P. aeruginosa* outer membrane protein OmpF triggers the effect on *rhlI*/*rhlR* and the enhancement in the production of the *rhl*-dependent *lecA* and pyocyanin. It is interesting to note that the effect of interferon gamma was detected during the late stationary phase of growth of PAO1 (high cell density) (Wu et al. 2005). Similarly, we detected the serum-induced enhancement in QS systems at the late stationary phase of growth of PAO1 (Figs. 2B, 3B and C). Other previously reported factors that enhance PAO1 QS systems are the circulating natriuretic peptides (NP). Blier et al. (2011) reported that NP, a family of eukaryotic hormones produced physiologically by atrial and endothelial cells, modulates the QS and QS-controlled factors in PAO1. Pretreatment of PAO1 with two of the peptides, the brain NP and C-type NP, significantly enhanced the production of 3OC_12_-HSL and C_4_-HSL, hydrogen cyanide, and exotoxin A (Blier et al. 2011). This effect appears to occur through the cAMP-activated Vfr and the *P. aeruginosa* global regulator PtxR (Blier et al. 2011). However, unlike our present findings, the C-type NP strongly inhibited pyocyanin production (Blier et al. 2011). Additionally, the NPs are produced by the host in response to certain stressful conditions such as bacterial infections and sepsis (Blier et al. 2011). In this study, the observed changes in the QS systems occurred in response to potential innate factors that exist in adult bovine serum (Figs. 2–4).

The clinical relevance of our findings is realized if one considers *P. aeruginosa* infected serum-containing tissues such as wounds. Within these localized infections, *P. aeruginosa* bacteria grow, reach a quorum, and communicate with each other through the autoinducer molecules. At this initial stage of quorum, and as part of the innate host defense, a potential serum factor(s) significantly reduces the synthesis of the autoinducer by repressing the autoinducer synthase genes (*lasI/rhlI*). However, as the infection progresses and the number of bacteria within the wound increase dramatically, this host defense may be overwhelmed. As a result and instead of being inhibited by serum, *P. aeruginosa* uses serum factors to enhance its virulence by increasing the production of different QS-controlled virulence factors.
